# Towards ubiquitous and nonintrusive measurements of brain function in the real world: assessing blink-related oscillations during simulated flight using portable low-cost EEG

**DOI:** 10.3389/fnins.2023.1286854

**Published:** 2024-01-08

**Authors:** Alexia Ziccardi, Kathleen Van Benthem, Careesa Chang Liu, Chris M. Herdman, Sujoy Ghosh Hajra

**Affiliations:** ^1^Department of Cognitive Science, Carleton University, Ottawa, ON, Canada; ^2^Department of Biomedical Engineering and Science, Florida Institute of Technology, Melbourne, FL, United States; ^3^Aerospace Research Centre, National Research Council Canada, Ottawa, ON, Canada

**Keywords:** blink-related oscillations (BRO), electroencephalography (EEG), aviation, cognitive state, mental workload, flight simulation, neuroergonomics, real world

## Abstract

Blink-related oscillations (BRO) are newly discovered neurophysiological phenomena associated with spontaneous blinking and represent cascading neural mechanisms including visual sensory, episodic memory, and information processing responses. These phenomena have been shown to be present at rest and during tasks and are modulated by cognitive load, creating the possibility for brain function assessments that can be integrated seamlessly into real-world settings. Prior works have largely examined the BRO phenomenon within controlled laboratory environments using magnetoencephalography and high-density electroencephalography (EEG) that are ill-suited for real-world deployment. Investigating BROs using low-density EEG within complex environments reflective of the real-world would further our understanding of how BRO responses can be utilized in real-world settings. We evaluated whether the BRO response could be captured in a high-fidelity flight simulation environment using a portable, low-density wireless EEG system. The effects of age and task demands on BRO responses were also examined. EEG data from 30 licensed pilots (age 43.37 +/− 17.86, 2 females) were collected during simulated flights at two cognitive workload levels. Comparisons of signal amplitudes were undertaken to confirm the presence of BRO responses and mixed model ANOVAs quantified the effects of workload and age group on BRO amplitudes. Significant increases in neural activity were observed post-blink compared to the baseline period (*p* < 0.05), confirming the presence of BRO responses. In line with prior studies, results showed BRO time-domain responses from the delta band (0.5–4 Hz) consisting of an early negative peak followed by a positive peak post-blink in temporal and parietal electrodes. Additionally, task workload and age-related effects were also found, with observations of the enhancement of BRO amplitudes with older age and attenuation of BRO responses in high workloads (*p* < 0.05). These findings demonstrate that it is possible to capture BRO responses within simulated flight environments using portable, low-cost, easy-to-use EEG systems. Furthermore, biological and task salience were reflected in these BRO responses. The successful detection and demonstration of both task-and age-related modulation of BRO responses in this study open the possibility of assessing human brain function across the lifespan with BRO responses in complex and realistic environments.

## 1 Introduction

Blinking has traditionally been considered an automatic physiological phenomenon that is associated with corneal lubrication. That is, the blinking-cycle helps initiate the tear film secretion of corneal lubrication (Doane, 1980, 1981; King-Smith et al., 2006; Braun and King-Smith, 2007). The physiological dynamics of the blink-cycle are intricate and have been thoroughly examined in the literature (Hung et al., 1977). However, the neurological mechanisms of the blinking-cycle are not well researched in relation to cognitive functioning. Yet, increasing evidence links blinking with various cognitive abilities. For example, in behavioral studies, changes in attentional demand or cognitive load show corresponding modulation of blink rates (Siegle et al., 2008; Oh et al., 2012; Wascher et al., 2015). Moreover, neuroimaging studies using fMRI have found links between spontaneous blinking and attentional mechanisms (Nakano et al., 2013). Investigation into the blink-cycle from a cognitive-neurological perspective is essential to understanding the role blinking can play in the daily lives of individuals and, in particular, during high-risk activities such as flying single-crew aircraft.

Approaches for understanding cognitive mechanisms, such as electroencephalography (EEG) (Nunez and Srinivasan, 2009; Hajra et al., 2018) which detects electrical activity from electrodes placed on specific areas of the human scalp to enable assessment of brain activity, typically treat blinks as a nuisance, with extensive efforts undertaken to denoise data and remove blink artifacts. However, a newly discovered neurophysiological phenomenon called blink-related oscillations (BRO) has been associated with spontaneous blinking (Liu et al., 2017). BROs are processes that demonstrate the brain’s increased rhythmic neural patterns, or oscillations, following spontaneous blinks (Bonfiglio, 2013, 2014; Liu et al., 2017; Liu, 2019; Liu et al., 2020a). The BRO has been found to originate from key brain regions such as the precuneus (Liu et al., 2017, 2019, 2020b) and surrounding areas in the parieto-occipital sulcus region (Bonfiglio, 2013). The morphology of a BRO response has been found to follow a two peak complex, whereby there is first a negative-going peak between 0 and 150 ms, followed by a positive-going peak (∼150–400 ms) (Bonfiglio, 2013, 2014; Liu et al., 2017, 2020b).

BRO activity has been shown to be present at rest and during tasks and is modulated by cognitive load and changes in sensory environment (Bonfiglio, 2013, 2014; Liu et al., 2017, 2020a,b; Liu, 2019). BROs are able to provide millisecond level information about brain functioning and can reflect changes in internal states (e.g., level of cognitive loading) and external environments (e.g., sensory environment), without requiring specific/complex tasks and setups such as those needed by traditional methods such as EEG-derived event related potentials (ERPs) (Luck, 2014). This creates the possibility for brain function assessments that can be integrated seamlessly into real-world settings using BROs. Therefore, the BRO phenomenon can provide insights into the investigation of cognitive functioning and should be considered an important area of study of cognition.

Prior studies of BROs were undertaken using sanitized laboratory environments, lab-based equipment (e.g., high-density EEG) and laboratory-centric tasks [e.g., comparison of 0-back vs. 3-back memory tasks (Hajra et al., 2021a)]. While these earlier studies provided the foundational knowledge necessary to understand and characterize BROs, there is a need to move toward real-world settings. That is, studies need to be conducted where features of the BRO can be explored with ecological validity. In studies of cognition, ecological validity allows for multiple cognitive abilities to be analyzed simultaneously, as participants are not limited to single tasks that are often controlled for testing some specific aspect of cognition. With natural environments, studying the relation between various cognitive factors and blink activity is possible and this allows for a more complete picture of how cognitive phenomena, captured by BROs, occur in real-world situations and environments. The next step in BRO research is therefore to evaluate and examine the BRO responses under the following conditions: (1) within complex naturalistic settings and across a range of age groups, (2) using tasks that are not limited to evaluation of a single cognitive domain but instead require multiple cognitive abilities, and (3) using technologies that enable the translation of BROs to the real world (e.g., easy-to-use, non-intrusive, portable EEG).

Aviation research is a domain with a long history of utilizing highly realistic complex high-fidelity simulated environments, making it an ideal avenue for the current research. Additionally, pilots are required to process a lot of information very quickly and efficiently, while flight tasks are cognitively demanding involving the coordination as well as the management of visual, tactile, memory, motor, and auditory demands, and fraught with risks of serious incidents or accidents (Bureau d’Enquetes et d’Analyses pour la Securite de l’Aviation Civile (BEA), 2002; Lenné et al., 2008; General Accounting Office, 2012; Government of Canada, 2018); thereby meeting the need of evaluating BRO responses when multiple cognitive domains are engaged. And, while with neurological studies, it is oftentimes the case that low-cost, portable EEG systems are viewed as challenging, it is crucial to examine BRO responses using such systems to enable deployment in the real-world settings. Portable, wireless, low-density EEG systems are not ideal, with respect to signal-to-noise ratios, since each electrode may receive proportionally more artifact information from various sources (muscle, cardiac etc.), as compared to high-density EEG systems (Duvinage et al., 2013). Despite the challenges with signal-to-noise ratios, portable and low-density EEG headsets offer advantages in the form of lack of intrusion and ease-of-use over laboratory high-density systems. For example, in the present study the portable wireless EEG system permitted pilots to move and behave naturally in the cockpit, similarly to how they would in an actual aircraft.

It is important to highlight that this choice of aviation as a demonstration domain was bidirectionally beneficial. That is, aviation provides a good backdrop to extend BRO research as highlighted above and synergistically may benefit from that as well since the aviation industry is in need of reliable physiology-based markers of cognitive state of pilots, driven in part by the safety impetus and in part by the desire to improve human-machine interaction, among other factors. A variety of techniques including subjective rating tools, behavioral assessments, and more recently physiology-based measurements are utilized in aviation to help better understand the cognitive and physiological state of pilots (Hajra et al., 2020b). For example, aviation as a domain has a long history of indexing mental workload states in real-world and simulated environments in both younger and older pilots (Wilson, 2002; Casto and Casali, 2013; Van Benthem et al., 2015; Causse et al., 2019; Dehais, 2019; Andrievskaia et al., 2020). However, BROs with their ability to provide objective brain-based measurements (as opposed to subjective rating scales), time-resolved assessments (as opposed to snapshots at certain time points), and lack of need for a secondary task (as is required for traditional techniques such as ERPs), may be uniquely positioned to help advance aviation cognition and human factors research.

Additionally, investigations of the effects of aging on safety have been particularly important in aviation research because of the disproportionate risk for accidents that is experienced by older pilots (Li et al., 2005). For example, the effects of older age on pilot performance and neural processes related to language were investigated using a 14-channel wireless EEG headset using a Cessna 172 flight simulator (Ziccardi et al., 2020; Fraser, 2021). In Ziccardi et al. (2020) older pilots showed poorer performance for key aviator tasks, and this was reflected in auditory event-related potentials (ERPs).

EEG has been used to explore the effects of various task features during simulated flight, more generally. For example, Dehais et al. (2019) demonstrated the effects of task demands on EEG time-frequency data using six dry-electrodes while pilots either flew or monitored simulated flight (Dehais, 2019). Studies have also utilized sparse EEG systems in virtual reality flight simulation. Researchers found neural correlates of mental workload and virtual hand representation in non-pilots by analyzing ERPs from neural information gathered using a 14-channel EEG system (Andrievskaia et al., 2020; Fraser, 2021).

The success of previous flight simulation research in detecting age and workload effects on neural processes using low-density EEG indicates that the data in the present research could also detect and show the effects of task modulations when collected using similar low-density EEG systems. In the present study, we evaluated whether BRO responses could be captured while younger and older licensed pilots flew in a flight simulator. It was hypothesized that the presence of BRO responses could be detected using a commercial, low-cost EEG device, and that the BRO response would be sensitive to task load manipulation and to pilot age, a salient biological factor.

## 2 Methods

### 2.1 Study background

The analyses outlined in this paper used data collected as part of a larger study investigating general aviation safety and human factors performed in the Advanced Cognitive Engineering Laboratory at Carleton University. Participants completed two experimental sessions, one involving a full-scale aircraft simulator with a broad angle display system and the other in a virtual reality flight simulator. The methods and findings discussed here are in relation to the first session, where the pilots were tasked with flying a route in the full-scale aircraft simulator. All participants provided written informed consent, and the study was approved by the university ethics committee operating under the Canadian Tri-Council Code of Ethics for psychological research.

### 2.2 Participant details

Licensed pilots were recruited from local pilot associations, flying schools, and clubs. Inclusion criteria included having a pilot permit or certification and a current medical certification. Pilots should also have flown as a pilot-in-command in the past 24 months prior to the study. As shown in Table 1, the sample included 30 pilots with varying levels of experience. Pilot age also varied (*X̄* = 43.37, *s* = 17.86). For analysis purposes, the participants were divided into younger (*n* = 17, aged 17–50 years) and older groups (*n* = 13, aged 51–70 years). The age group threshold for “younger and older” designations were based on commonly used demarcations in literature (Van Benthem et al., 2015) and were optimized for group sizes.

**Table 1:** Pilot-relevant information.

	Range	Minimum	Maximum	Sum	Mean	Standard deviation
Age	52	18	70	1,301	43.37	17.86
Total flight hours	11,998	2	12,000	31094.6	1036.49	2380.24
Total years licensed	69	1	70	374	12.47	14.39
Pilot level	5	1	6	111	3.7	1.49

*N* = 30. Pilot level was ranked as follows; 1 – Student, 2 – Recreational, 3 – Private Pilot (PPL) no extra rating, 4 – PPL extra ratings, 5 – PPL with IFR or commercial, 6 – Airline Transport (ATP).

### 2.3 Task details

#### 2.3.1 Briefing

The researchers guided the pilots through an introductory PowerPoint presentation in order to familiarize the participants with the study purpose, the task requirements, as well as the equipment and scenarios used in the experiment. The briefing presentation contained detailed information about the planned flight route, including altitudes, headings, and airspeeds for each leg of the outbound and inbound segments of the route.

#### 2.3.2 Materials

**Figure 1:**
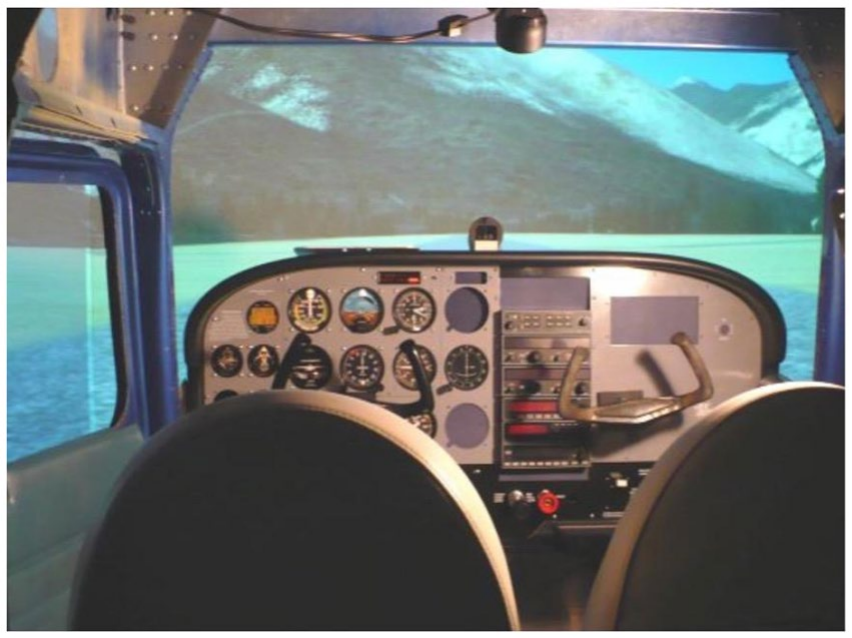
Cessna 172 simulator cockpit controls and visual displays.

Pilots flew in a Cessna 172 Level 6 Flight Training Device. The flight control unit included key instruments, a yoke, throttle, and flaps (see Figure 1). For navigation material, traditional paper charts, as well as a digital navigational aid (the ForeFlight Type B Electronic Flight Bag) were provided. Participants wore two biometric recording devices, an electroencephalography (EEG) headset and the Empatica E4 biometric wristband to record physiological states (e.g., skin temperature and conduction, and heart rate). Since the focus of this paper is on the neurophysiological phenomenon of BROs, the data collected using the wristband is not reported herein. The EEG data was collected via the EMOTIV EPOC+ 14-channel wireless system. To record the EEG data, the EMOTIV software TestBench was used, applying a bandwidth of 0.2 to 45 Hz before further processing in EEGLAB, an open-source software running on MATLAB v. R2022a.

#### 2.3.3 Flight task design

**Figure 2:**
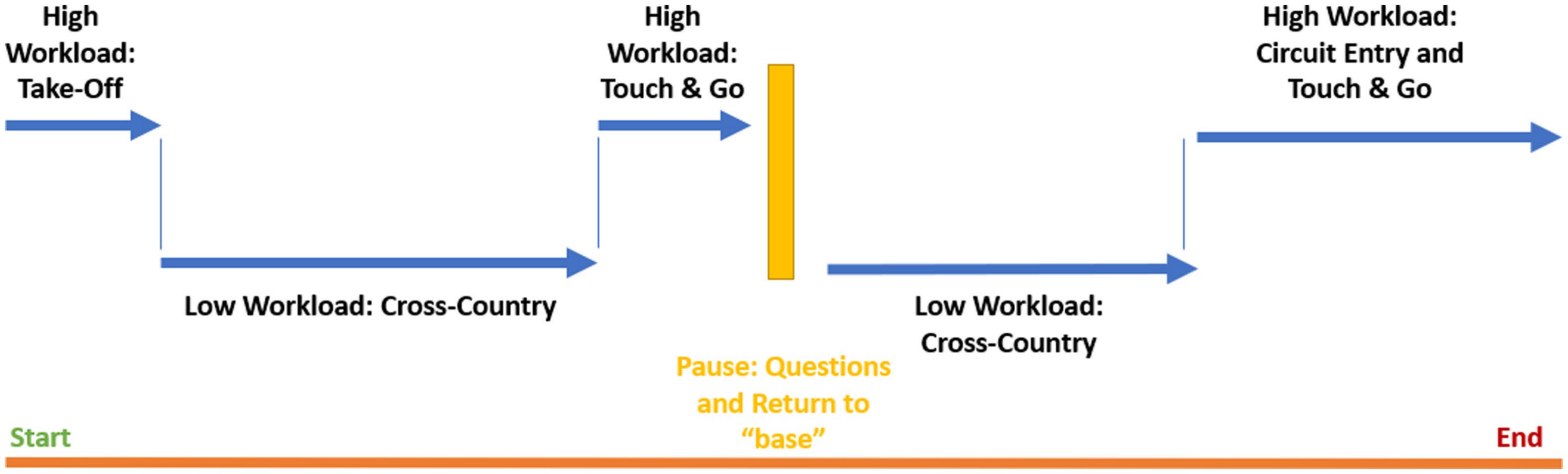
Segmented workload conditions.

Participants flew practice circuits in terrain similar to the experimental conditions before beginning the experiment so that they were familiar with aircraft handling and the tasks to be performed. The route began at a small general aviation aerodrome, heading westerly along two rivers. After 10 min of complex flight at lower altitudes over hilly terrain, the route followed a river and was flown at higher altitudes. After 10 min of this easier flight the pilots were required to complete a “touch and go” maneuver, where they touch down briefly and immediately take off again, at another small general aviation aerodrome. The return flight followed this route but in the opposite heading, for a return to the departure aerodrome. The total duration of the flight was approximately 60 min. The segmentation of the route into low and high workload conditions is detailed in Figure 2.

#### 2.3.4 General flight tasks

During the flight, air-to-air radio messages were broadcasted from local pilots. Participants were instructed to listen for these messages as they were asked questions about their content at the pause between the two main segments of the flight and at the end of the flight. The messages were presented in earbuds that the pilots wore during the flight. There were 14 radio messages in total, which contained information about other aircraft flying simultaneously (e.g., other pilot’s aircraft type, call sign, location, and intention). Additionally, throughout the flight pilots also heard tones played randomly that they responded to via a thumb switch. After completing a touch and go at the second aerodrome, experimenters paused the flight and participants were asked to indicate where other aircraft were located on a map and report on the other details of the radio messages heard (for more information, see Ziccardi et al., 2020, 2021). At the end of each leg of flight, pilots located other aircraft positions on a map and reported on the radio call details from ground services and from other aircraft.

### 2.4 Data acquisition

Electrical potentials on the scalp were recorded using the EMOTIV EPOC+ 14 channel wireless EEG system. This commercial system has been shown to be robust and capable of recording EEG relevant to studies of cognition and aviation (Knoll et al., 2011; Khan et al., 2017; Bollock et al., 2019; Binias et al., 2020). Accessible electrodes were labeled as AF3, F7, F3, FC5, T7, P7, O1, O2, P8, T8, FC6, F4, F8, AF4, with the channel placements following the international 10–20 system. The electrical potentials were referenced to electrodes P3 and P4 by the EPOC+ system using proprietary deferential montage methods where the P3 electrode was used as the Common Mode Sense (CMS) and the P4 electrode was used as the Driven Right Leg (DRL). The EEG recordings were collected at 2048 Hz, and then downsampled to 256 Hz and were transmitted wirelessly via Bluetooth to an iMac desktop computer using the TestBench software. Further downsampling to 128 Hz was conducted in EEGLAB (Delorme and Makeig, 2004). EEG calibration involved applying saline solution to the felt pads on each electrode and monitoring the signal quality, assuring impedance levels remained in the 10–20 kΩ range. Signal quality was measured throughout the experiment. After the first leg of the flight, saline was re-applied if an electrode was found to be noisy. A bandwidth filter of 0.2 to 45 Hz was applied before further processing using the open source EEGLAB running on MATLAB v. 2019b (Delorme and Makeig, 2004).

### 2.5 Data processing details and extraction of BRO

Raw EEG data was decomposed using a preliminary independent component analysis (ICA), to extract individual components from the mixed signals, many of which were considered noise artifacts. Labels were inserted into the data to classify the beginning and end of the first and second leg of the flights and the low and high workload segments of the flight. The Blinker v 1.1.2 (Kleifges et al., 2017) plugin for EEGLAB was used to identify blinks in the continuous EEG data. The Blinker software was also used to identify blink peak latencies and peak values (Kleifges et al., 2017). Blinker is a customizable tool that extracts data related to blinks and their properties. The Blinker software creates an optimal blink template based on all potential blinks, and then provides output that identifies the number of blinks, the number of acceptable blinks, and the latencies of key blink features, such as the blink peaks, which were used in this experiment. Good blinks show an acceptable level of fit with the optimal blink template derived from the data (see Kleifges et al., 2017 for more information). For this study, the parameters used for extracting the blinks included the AF3 electrode for signal acquisition, filtering with a low cut-off of 1 hz and a high cut-off of 20 hz and an acceptable blink amplitude range of 3–50 μV.

**Figure 3:**
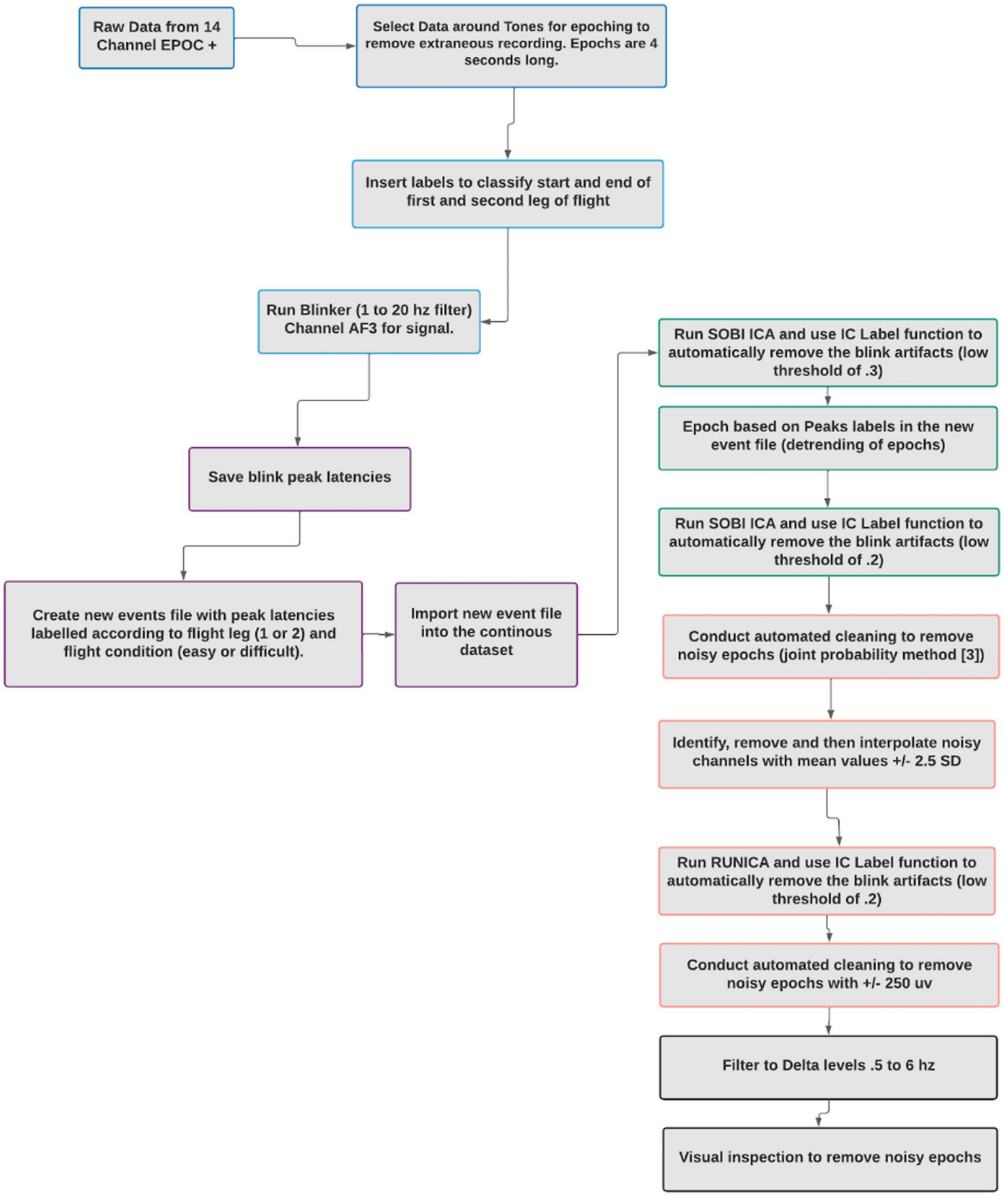
Artifact denoising procedure.

New EEG event files were created with peak latencies labeled according to flight leg (1 or 2) and flight condition (high workload or low workload). The continuous data was subjected to ICA decomposition and then EEGLAB’s IC Label function, which automatically removed blink artifacts with a low certainty threshold of 30%. Major sources of noise, including muscle, heart, line, and channel noise were identified and removed from the data. The blind source separation method for ICA decomposition was SOBI. SOBI is an algorithm known to work well at separating data extracted from sparse electrode data sets and is efficient at detecting ocular artifacts (Joyce et al., 2004; Choi et al., 2005). The data was then epoched into 2-s segments based on each blink peak latency, as per the new event files. An automated cleaning protocol was conducted to automatically remove epochs with +/− 500 μV. Epochs that contained a blink within 1.5 s of another blink were removed to prevent blink-to-blink contamination. Noisy channels were identified and removed based on a threshold of mean values of +/− 2.5 SD. The removed channels were interpolated using EEGLAB. ICA and the IC Label function were conducted to automatically remove blink artifacts, again with a low probability threshold of 0.2. A delta frequency band filtering was conducted (0.5 to 6 hz). A final visual inspection of each epoch was conducted by a researcher to remove any remaining noisy epochs. These processing steps are detailed in Figure 3.

When investigating BROs, it is essential to remove ocular artifacts around the blink peaks, to avoid confusing ocular contamination for brain-related processes. To confirm the removal of blink artifacts during the data cleaning process, an Ocular Contamination Index (OCI) was generated for each participant (as per Liu et al., 2020a). To generate the OCI, the amplitude variance across all channels were calculated pre-and post-cleaning at both the 0 ms point (where a blink artifact should appear) and a baseline point, −700 ms. The amplitude variance across these channels was then used to create a ratio of variance between the 0 point and baseline point.

**Figure 4:**
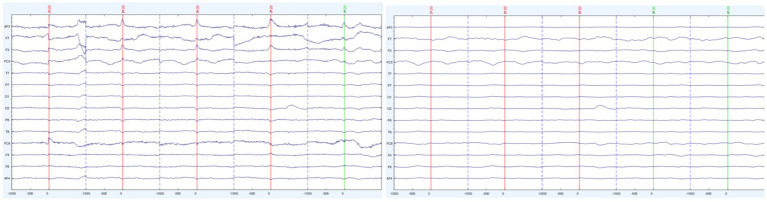
Sample participant’s segmented trials before (left) and after (right) artifact removal.

**Figure 5:**
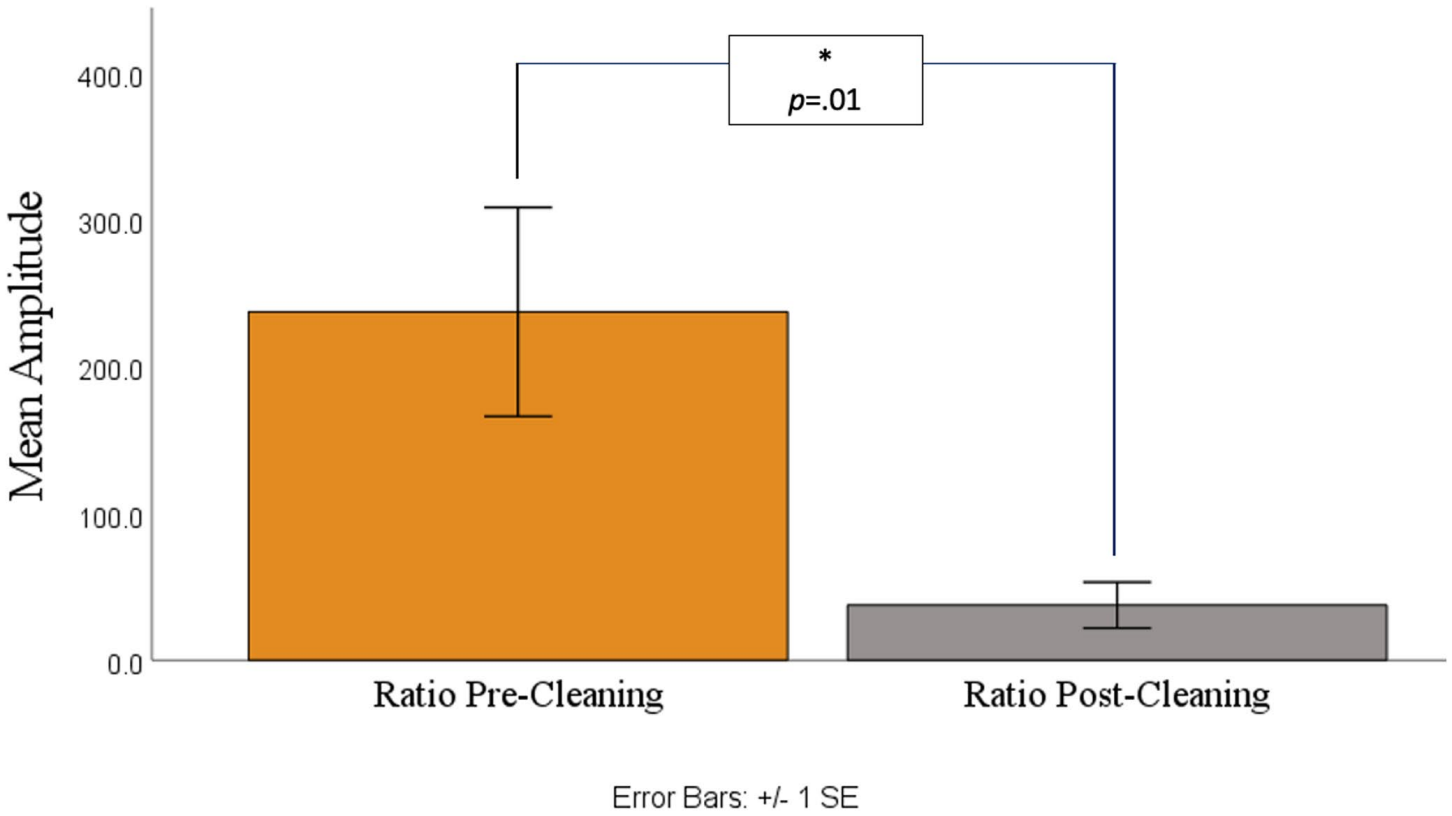
Ratio of amplitude variance between the 0 point and baseline point before (left) and after (right) cleaning.

A paired *t*-test was then conducted for analyzing pre-cleaning and post-cleaning ratio values. As shown in Figures 4, 5 there was a significant difference between the pre-cleaning ratios (*M* = 237.26, SD = 389.93) and post-cleaning ratios (*M* = 37.42, SD = 85.82), *t*(29) = 2.71, *p* = 0.01, indicating only trace ocular contamination remaining in the cleaned datasets.

### 2.6 Analyses

#### 2.6.1 Validation of the BRO

To test whether the BRO responses could be detected using low-cost EEG headset systems, pairwise sample t-tests were conducted, at each electrode for the younger participants in the low workload conditions, in order to approximate previous research. Younger participants were of specific interest as there is little research on BROs using the older subjects’ age range and therefore, what a BRO response looks like in older subjects is not known. Therefore, the validation analyses had a reduced sample size (*n* = 17). Moreover, the low workload conditions were of particular interest as previous research has shown BRO responses to be the most evident in lower workload conditions (Liu et al., 2020b) and only tested on younger groups of participants. The comparisons of baseline versus BRO components were conducted using the pre-blink baseline (−900 ms to −725 ms) window and C1 and C2 intervals (50 ms to 150 ms for C1 as well as 151 ms to 400 ms for C2). Based on findings, an *ad hoc* analysis, exploring latency effects for age and workload was conducted.

#### 2.6.2 Evaluation of age and workload effects

**Figure 6:**
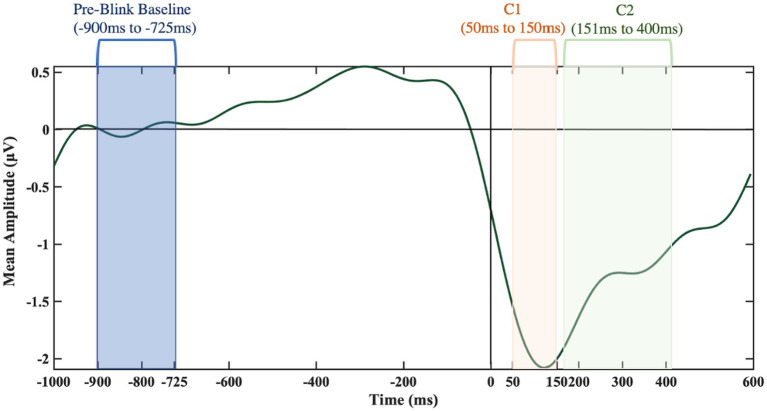
Key time frames of interest from the T8 electrode.

Using the Study function in EEGLAB, grand averages of the BRO responses were created across the 30 participants. Using the EEGLAB Darbaliai plugin, mean amplitudes were extracted for the latencies of interest. Specific latency ranges were selected for a pre-blink baseline (−900 ms to −725 ms), and the two post-blink peak intervals: 50 ms to 150 ms for first negative going deflection as well as the 151 ms to 400 ms peak interval for the second positive going deflection (see Figure 6). The extraction process was repeated for electrodes in the temporal (T7 and T8) and parietal brain regions (P7 and P8 electrodes). The brain regions of interest were temporal and parietal regions. Previous literature has identified BROs using central electrodes (Liu et al., 2017, 2019, 2020b). The EEG system used in this study does not have a midline electrode, so the best proxy electrodes were used. The occipital regions were not analyzed as this system produces excessive noise in occipital electrodes, mostly due to head movement and therefore, the parietal and temporal electrodes were used. In addition, the parietal and temporal electrodes most closely resemble the electrodes used in the seminal BRO papers.

## 3 Results

### 3.1 Blink behavior

To assess the blink behavior of participants, the Blinker plugin identified standard morphological blink features as well as what data from the signal were considered blinks. Blinker identified the total blinks per minute, the overall total number of blinks, and “good” blinks, which indicated the ratio of blinks meeting the certainty threshold (a linear correlation of greater than 0.9). Table 2 shows the results of the blink analysis. The good blinks were used as a reference to identify peaks and extract BRO properties and were therefore of particular importance in the present work.

**Table 2:** Number of blinks per participant.

	Minimum	Maximum	Mean	Standard deviation
Blinks per minute	3	22	13.2	4.46
Total number of blinks	458	2,372	1443.47	403.57
Total number of good blinks detected	180	1,860	977.27	347.3

*N* = 30. Good blinks refer to blinks that had the appropriate signal-to-noise ratio (non-negative scalar between 0 and 1) (see Kleifges et al., 2017).

To examine the effects of age and workload on BRO responses, a mixed model ANOVA, with one within-subject effect (workload) and one between-subject (age group), was conducted with flight hours as a covariate. Additional analyses included mixed model ANOVAs with the same factors and the number of blinks as a covariate to account for any differences in SNR due to the unequal number of trials (i.e., blinks) across conditions. In all cases no significant effect of blink number was found, *p* > 0.05.

Table 3 shows the results of the features regarding the blinks’ morphology for both age groups. The blink morphology parameters pertain to maximum peak amplitudes or the blink duration (total, half, or from left to right baseline), and the steepness of the ascending and descending slopes (see Kleifges et al., 2017 for information on the blink shape parameters). To examine the effects of age on the blinks’ morphology, one-way ANOVAs were conducted on the blink variables with age group as the between-subject variable.

**Table 3:** Blink morphology features.

Blink variable	Age group	Mean	Standard deviation	Statistic
Median amplitude of the best candidate blinks	Younger	175.47	57.96	*F*(1,29) = 0.23, *p* = 0.64
	Older	186.89	72.85	
Positive amplitude velocity	Younger	4.62	0.57	*F*(1,29) = 0.16, *p* = 0.70
	Older	4.53	0.62	
Negative amplitude velocity	Younger	7.15	0.86	*F*(1,29) = 0.09, *p* = 0.77
	Older	7.06	0.71	
Blink duration (left half)	Younger	0.12	0.02	*F*(1,29) = 0.12, *p* = 0.73
	Older	0.12	0.03	
Total blink duration (rightBase – leftBase)/srate	Younger	0.3	0.03	*F*(1,29) = 0.55, *p* = 0.46
	Older	0.3	0.03	
Blink duration at half of the maximum amplitude	Younger	0.1	0.02	*F*(1,29) = 0.003, *p* = 0.96
	Older	0.1	0.02	
Blink tent duration	Younger	0.19	0.03	*F*(1,29) = 0.007, *p* = 0.93
	Older	0.19	0.04	
Blink duration	Younger	0.22	0.02	*F*(1,29) = 0.02, *p* = 0.88
(rightZero – leftZero)/srate	Older	0.22	0.05	

Durations are in seconds. Positive amplitude velocity refers to the slope of the positive-going segment of the blink component, whereas negative amplitude velocity refers to the slope of the negative-going segment of the blink component.

### 3.2 Validation of the BRO

For each channel of interest, comparisons between the pre-blink baseline amplitude and each of the post-blink peaks (C1 and C2) amplitudes were undertaken to confirm the presence of the characteristic BRO features. Only the younger group (low workload condition) was used in the BRO validation to aid in comparison with known BRO features, as previous work that characterized the BROs was conducted with participants under 50 years of age. The distributions of the relevant amplitudes at the electrode sites were primarily normal as confirmed with the Q-Q plot inspections and paired *t*-tests were utilized to compare the means.

#### 3.2.1 Parietal electrodes

**Figure 7:**
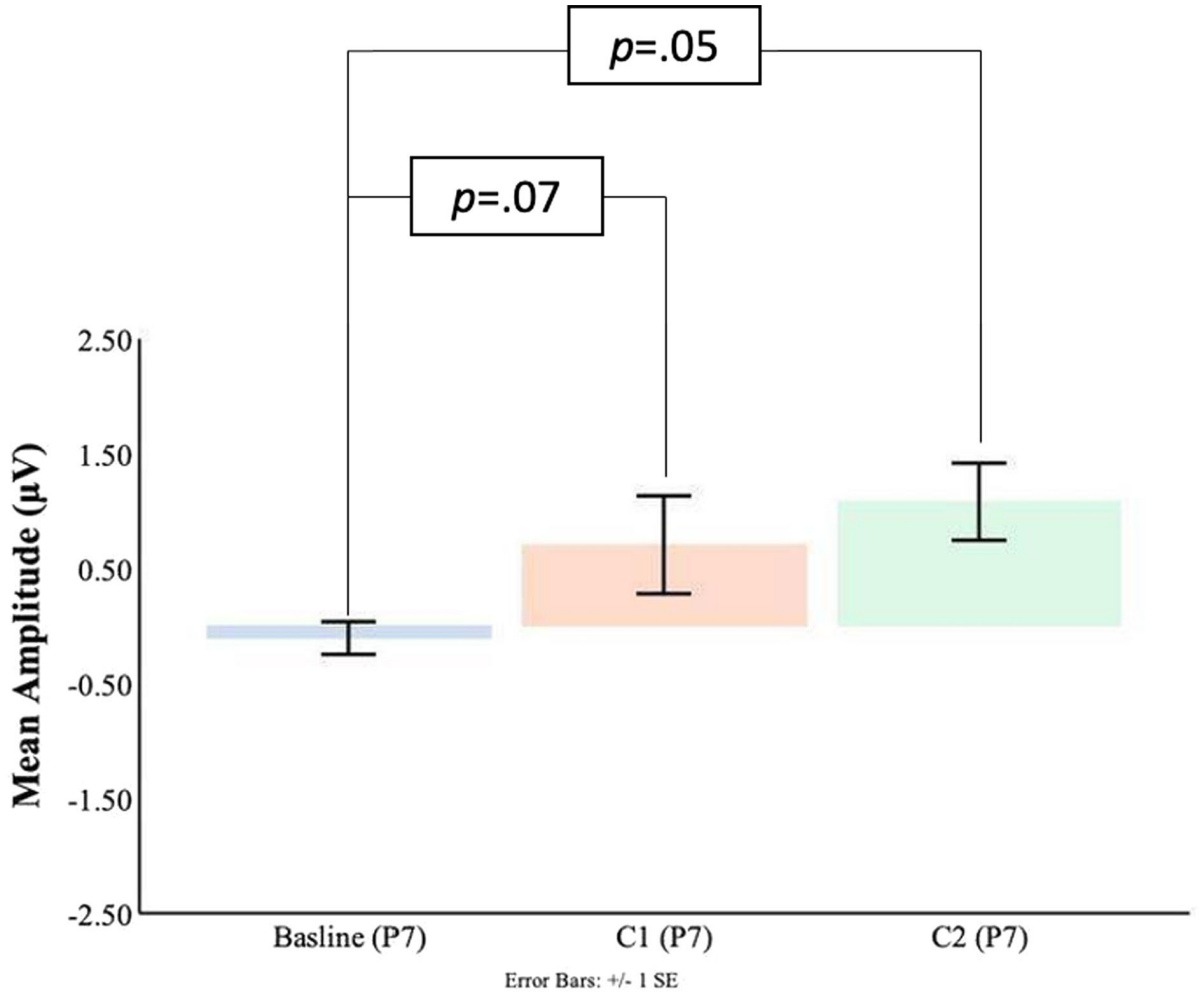
C1-C2 components baseline comparisons for the P7 electrode.

**Figure 8:**
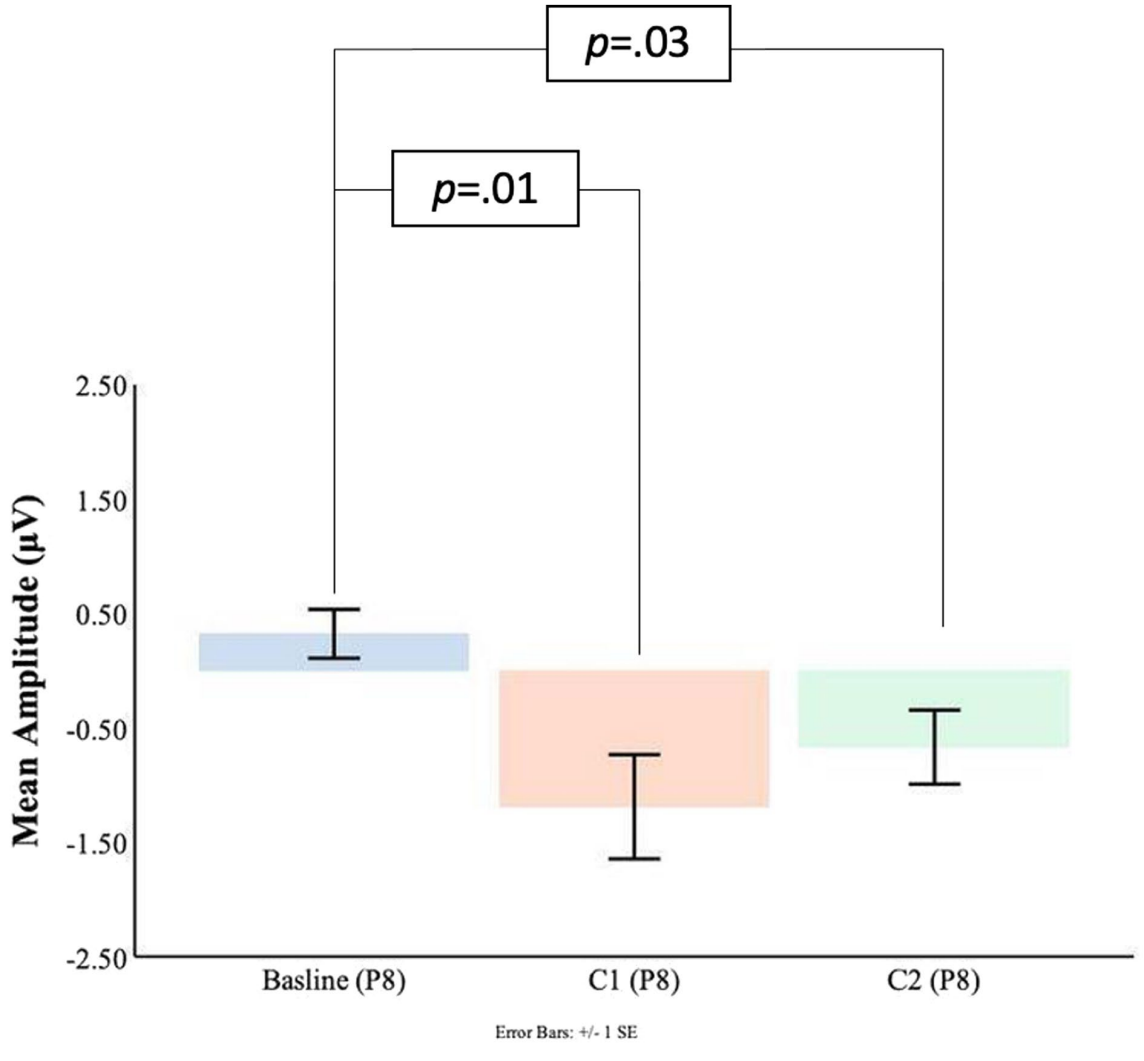
C1-C2 components baseline comparisons for the P8 electrode.

As shown in Figures 7, 8 at the P7 electrode the C1 mean amplitudes were larger (*M* = 0.71, SD = 0.76) than the pre-blink baseline mean amplitudes (*M* = −0.10, SD = 0.58), and this trended toward significance *t*(16) = −1.58, *p* = 0.07. There was a significant difference between the pre-blink baseline mean amplitudes and the C1 mean amplitudes in the P8 electrode such that the C1 mean amplitudes were larger (*M* = −1.19, SD = 1.88) than the pre-blink baseline mean amplitudes (*M* = 0.32, SD = 0.88), *t*(16) = 2.82, *p* = 0.01.

Significant differences were also found for the C2 mean amplitude comparisons to the pre-blink baseline mean amplitudes. In the P7 electrode, the pre-blink baseline mean amplitude (*M* = −0.10, SD = 0.58) was significantly lower than the C2 mean amplitude (*M* = 1.08, SD = 1.39), *t*(16) = −2.90, *p* = 0.05. The P8 electrode also showed that the pre-blink baseline mean amplitude (*M* = −0.67, SD = 1.34) was significantly lower than the C2 mean amplitude (*M* = −1.19, SD = 1.88), *t*(16) = 2.06, *p* = 0.03.

#### 3.2.2 Temporal electrodes

**Figure 9:**
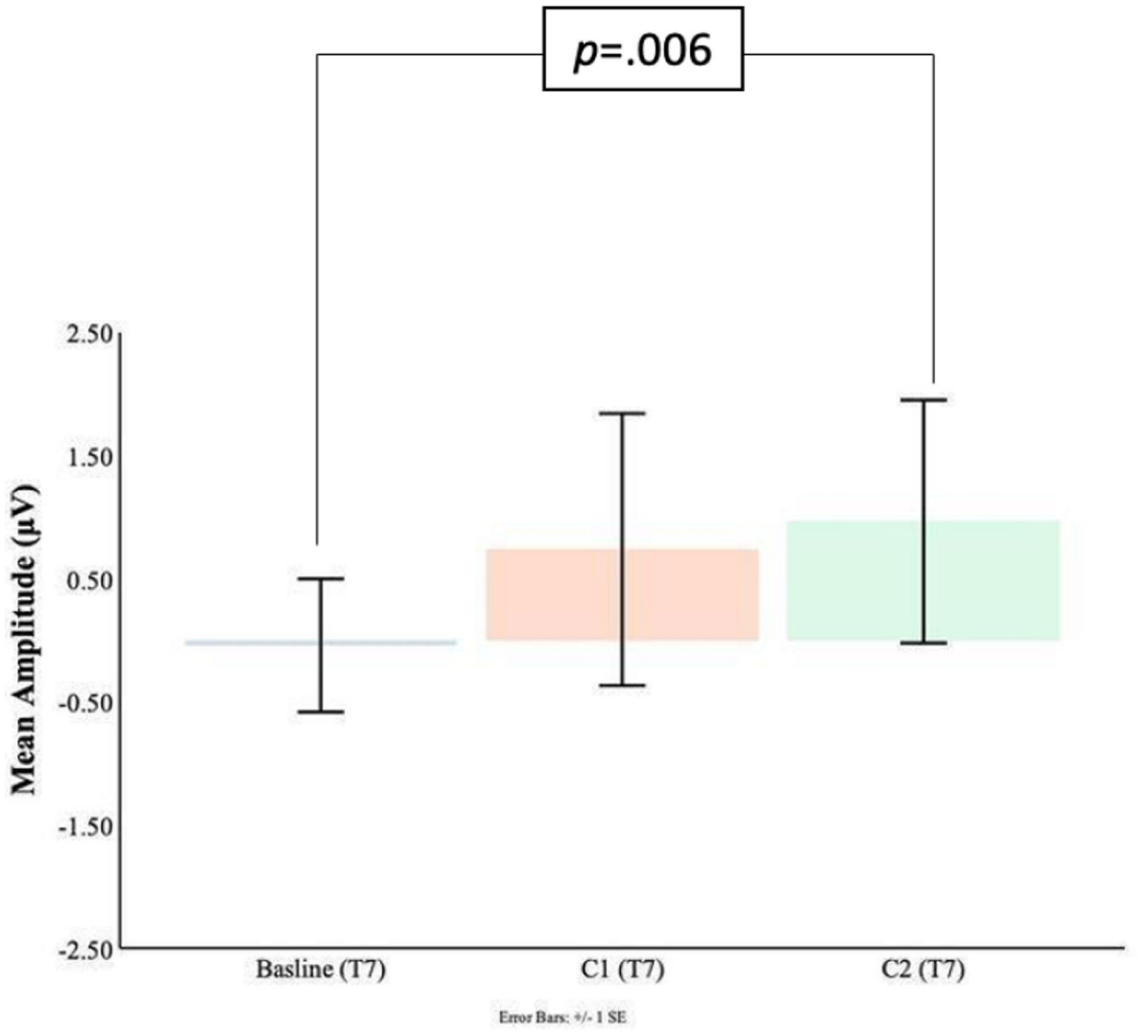
C1-C2 components baseline comparisons for the T7 electrode.

**Figure 10:**
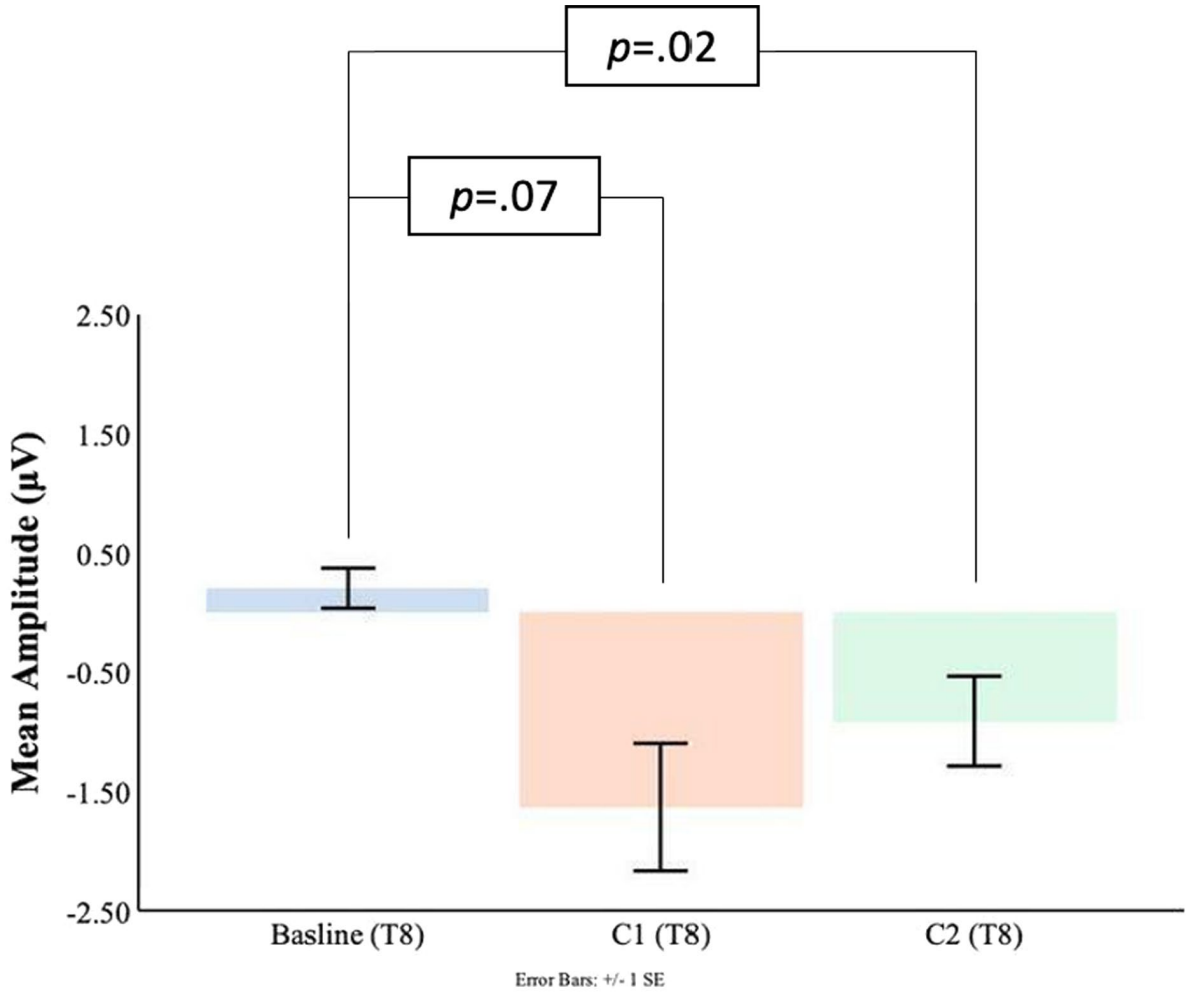
C1-C2 components baseline comparisons for the T8 electrode.

Comparisons between the pre-blink baseline mean amplitudes and C1 mean amplitudes were conducted in the temporal electrodes. It was found that in the T8 electrode, the C1 mean amplitudes were significantly larger (*M* = −1.63, SD = 2.21) than the pre-blink baseline mean amplitudes (*M* = 0.21, SD = 0.69), *t*(16) = 2.87, *p* = 0.006. No significant difference was found between the pre-blink baseline mean amplitudes and C1 mean amplitudes in the T7 electrode. Please refer to Figures 9, 10.

Significant differences were also found for the C2 mean amplitudes comparison to the pre-blink baseline mean amplitudes. In the T7 electrode, the pre-blink baseline mean amplitudes (*M* = −0.04, SD = 1.11) were lower (trending toward significance) than the C2 mean amplitudes (*M* = −0.96, SD = 2.03), *t*(16) = −1.56, *p* = 0.07. The T8 electrode also showed a significant difference such that the C2 mean amplitudes were significantly larger (*M* = −0.91, SD = 1.55) than the pre-blink baseline mean amplitudes (*M* = 0.21, SD = 0.69), *t*(16) = 2.34, *p* = 0.02.

### 3.3 Age and workload effects

#### 3.3.1 Parietal electrodes

**Figure 11:**
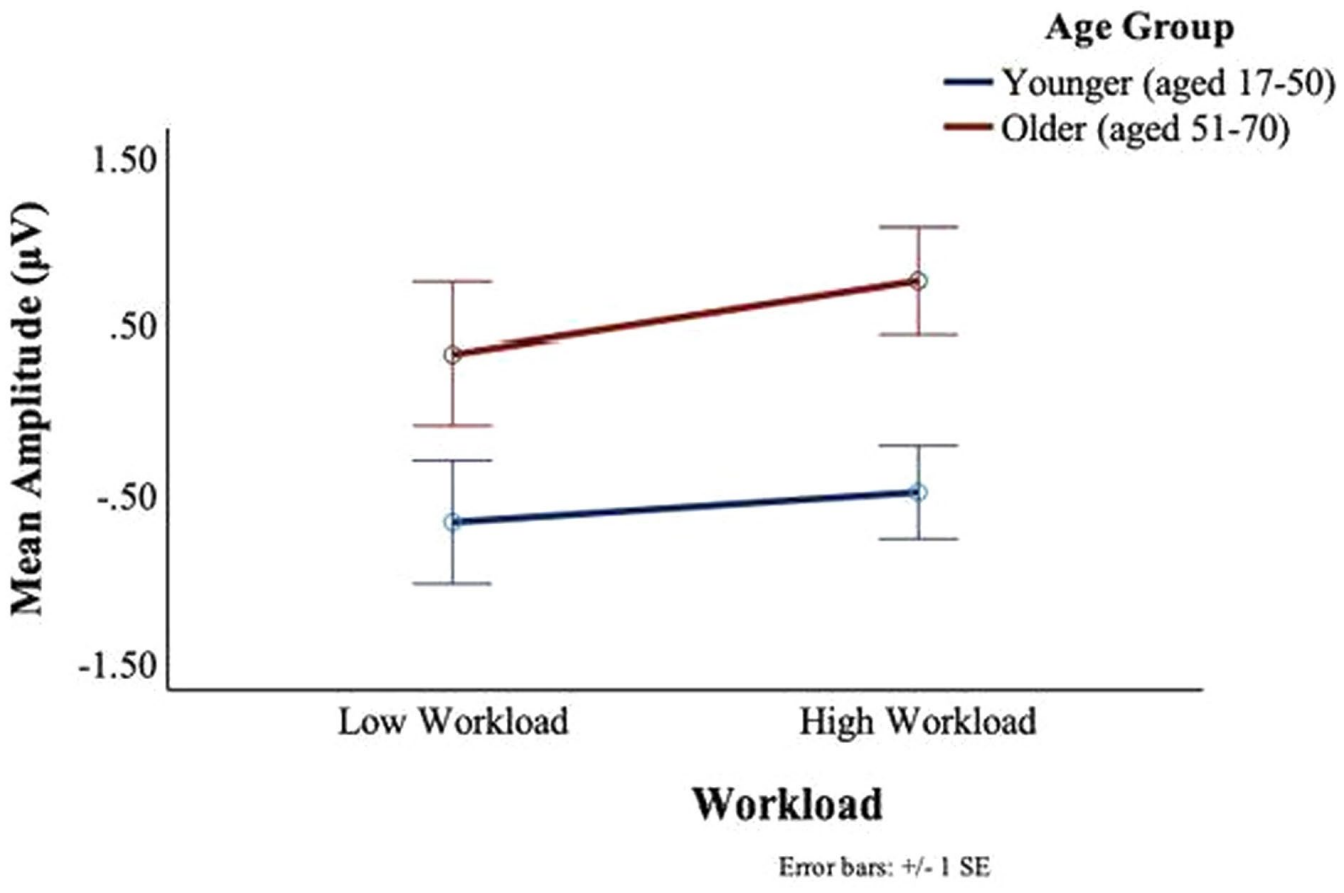
BRO C2 component: workload and age effects.

No main or interaction effects of age or workload were found in the P7 electrode for neither the C1 nor the C2 deflections. For the P8 electrode, no main or interaction effects of age or workload were found for the C1 deflection. However, for the C2 deflection, there was a main effect of workload, *F*(1,27) = 4.09, *p* = 0.05, *ηp2* = 0.13, such that in the low workload condition participants showed greater C2 deflections (*M* = −0.23, SD = 1.51) than in the high workload conditions (*M* = 0.05, SD = 1.25). Moreover, there was a main effect of age, *F*(1,27) = 5.35, *p* = 0.03, *ηp2* = 0.17, with the younger group staying predominantly below the baseline (*M* = −0.58, SD = 1.08), and the older participants remaining above the baseline (*M* = 0.54, SD = 1.49) (see Figure 11).

**Figure 12:**
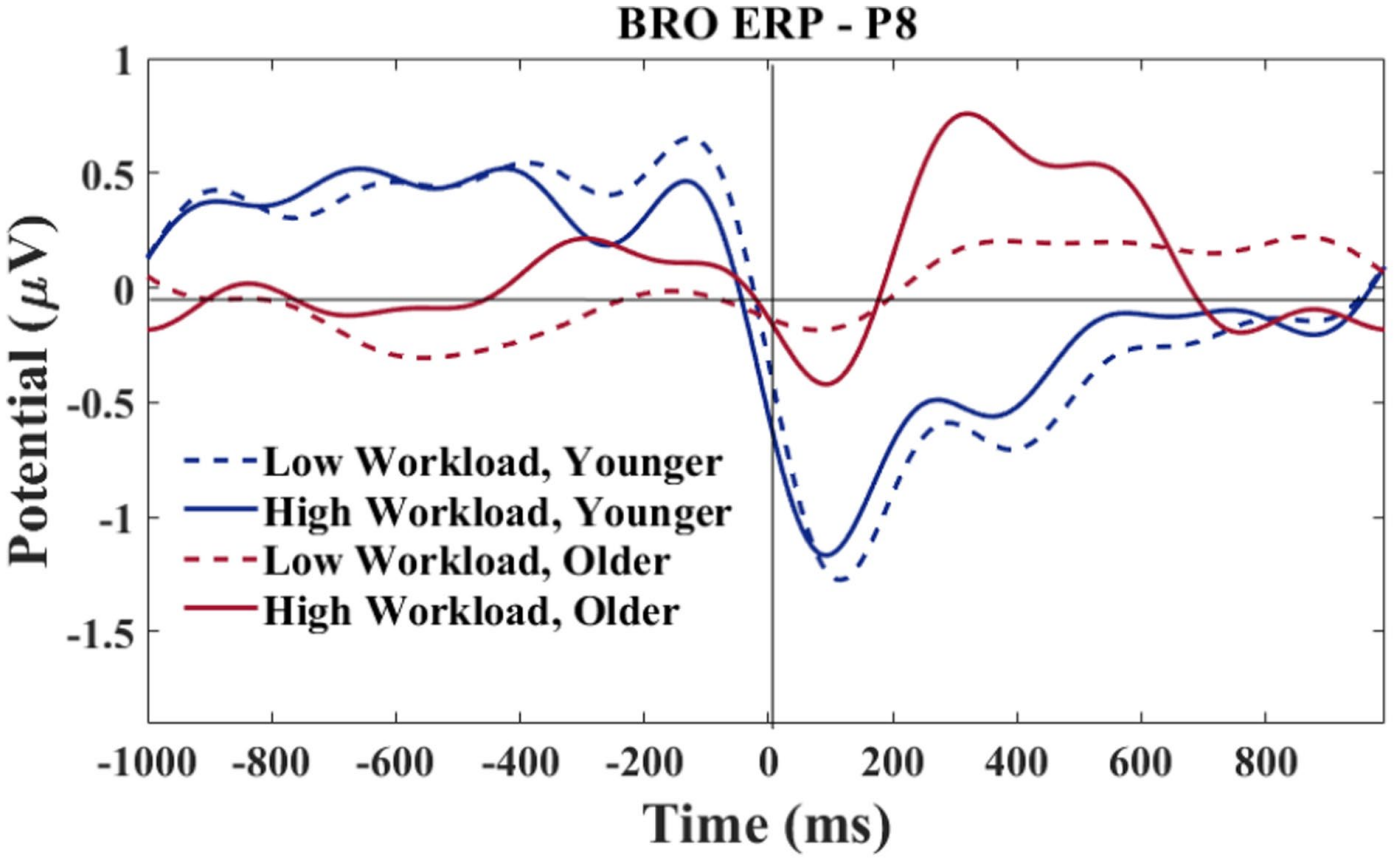
P8 electrode-BRO for age and workload. Waveforms smoothed using a frequency filter of 7 Hz and individual files removed where any of the waveform amplitude values exceeded 5 μV.

A grand average waveform for the right parietal electrode (P8) was produced to investigate the latencies and waveforms associated with the BRO. As shown in Figure 12, the younger group showed similar BRO morphology regardless of workload condition. In contrast, the older group had marked attenuation at the C1 latency but significantly greater positive deflection at C2.

#### 3.3.2 Temporal electrodes

**Figure 13:**
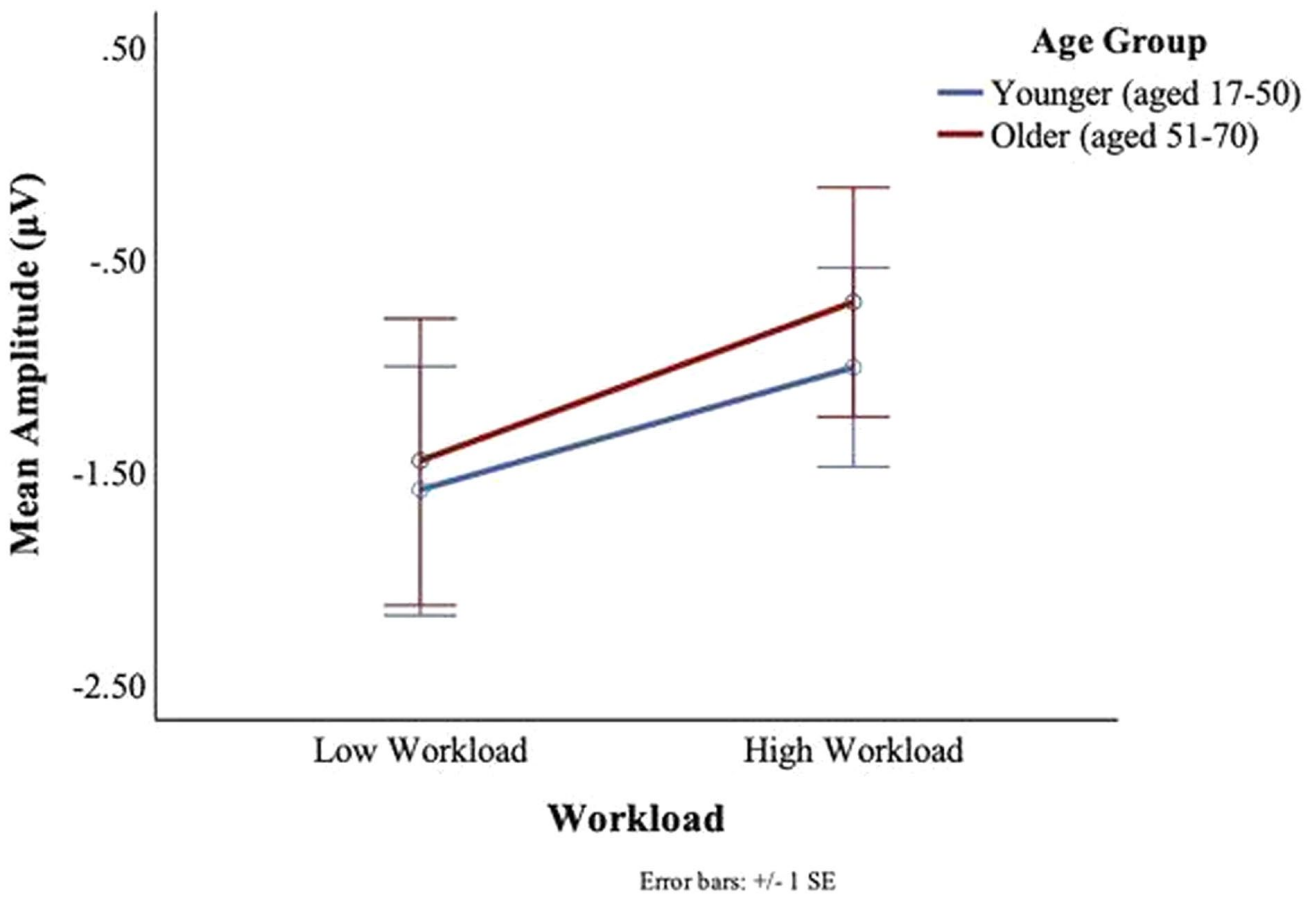
BRO C1 component workload and age effects (T8).

No main or interaction effects of age or workload were found at the T7 electrode for the C1 or C2 deflections. For the T8 electrode, there was a main effect of workload for C1, *F*(1,27) = 5.025, *p* = 0.03, *ηp2* = 0.16, such that larger signal amplitudes were observed in the low workload conditions (*M* = −1.53, SD = 2.27) as compared to the high workload conditions (*M* = −0.88, SD = 1.89) (see Figure 13). There was no significant main effect of age or interaction between age and workload on the C1 amplitudes of the BRO response at T8.

**Figure 14:**
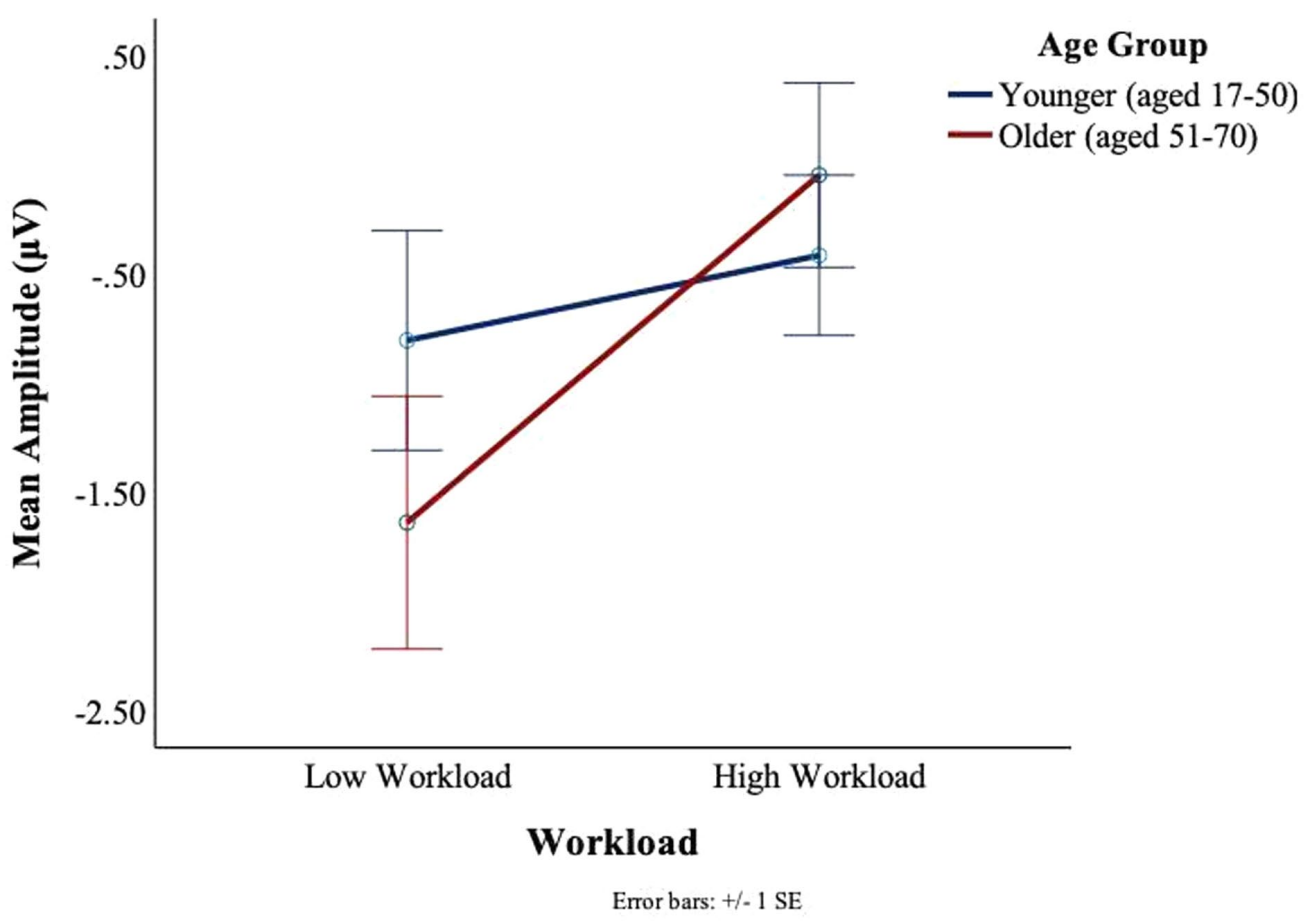
BRO C2 component workload and age effects (T8).

There was a main effect of workload in the T8 electrode for C2, *F*(1,27) = 11.41, *p* = 0.002, *ηp2* = 0.3, such that greater deflections were observed in low workload conditions (*M* = −1.17, SD = 2.00) as compared to the high workload conditions (*M* = −0.26 SD = 1.44) (see Figure 14). An interaction trend was found between age and workload, *F*(1,27) = 3.76, *p* = 0.06, *ηp2* = 0.12. The interaction effect was such that in the younger group there was little impact of workload on the BRO amplitude. In contrast, some workload effects could be seen in the older group, where the mean amplitudes were attenuated (*M* = −1.50, SD = 2.50) in the low workload as compared to the high workload (*M* = −0.3, SD = 1.61) (see Figure 14).

**Figure 15:**
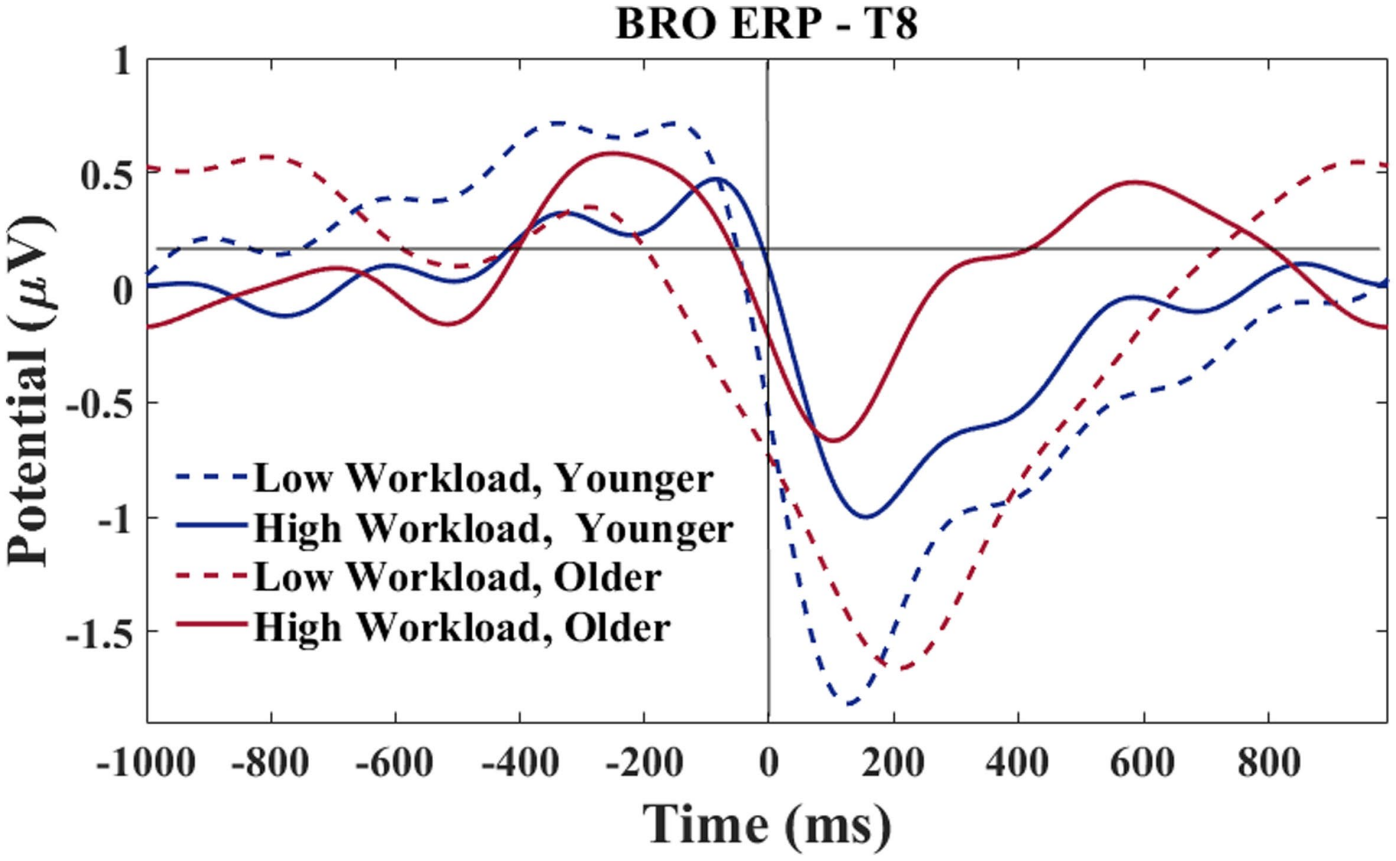
T8 electrode-BROs for age and workload. Waveforms smoothed using a frequency filter of 7 Hz and individual files removed where any of the waveform amplitude values exceeded 5 μV.

A grand average waveform for the right temporal electrode was produced to investigate the latencies and waveforms associated with the BRO. As shown in Figure 15, at the C1 latency both the younger and older group had greater negative-going deflections in the low workload as compared to the high workload. At the C2 latency only the older group in the high workload condition achieved a positive going deflection that was above the baseline.

### 3.4 BRO response latency analysis

Following the results of section 3.2.2, a morphological difference between the workload conditions can be observed for the T8 electrode (see Figure 15). The difference is such that the high workload condition showed a 2-peak C1-C2 complex for both older and younger groups. In contrast, the low workload condition only showed a 2-peak complex for the younger group, while the older group exhibited a single peak morphology. Additional analyses were conducted to explore the relationship of the peaks with age.

Based on the morphology of the BRO responses illustrated in Figure 15, the time window of interest for this analysis was specified as 50–250 ms. The amplitude values associated with the largest negative going deflection within the time window were recorded by two separate investigators. Only participants who showed a clear negative going deflection within the specified time window were considered for this analysis. Since visual observation was required for determining the amplitude of each peak, an inter-rater reliability analysis was done to confirm the accuracy of the amplitude values extracted by both researchers. The single measure intraclass correlations for the LWL values was 0.96 and 0.99 for the HWL. In the rare case of rater disagreement, consensus was achieved via discussion.

A mixed model ANOVA, with one within-subject effect (workload) and one between-subject effect (age group), was conducted on the C1 values extracted. No significant effect of workload was found for the C1 peak latency, *F*(1,22) = 2.28, *p* = 0.15, *ηp2* = 0.09. Furthermore, no significant interaction between workload and age was found for the C1 peak latency either, *F*(1,22) = 2.95, *p* = 0.10, *ηp2* = 0.12.

## 4 Discussion

Investigating BRO responses in more realistic settings using a low-density EEG system can be an effective way to understand the cognitive factors associated with blinking. The present research investigated whether a low-density EEG system can detect BRO responses in a realistic, and cognitively demanding flight environment. A secondary objective of the study was to investigate the effects of task manipulation (workload) and biological salience (age) on BRO responses. Results showed that BRO responses were identified in temporal and parietal brain regions and both workload and age effects were evident in the captured BRO responses.

### 4.1 Verification of the BRO response

Results showed that the post-blink neural responses in the present work had similar characteristics as reported in previous studies that were conducted using controlled, laboratory environments. The BRO was verified by comparing mean amplitudes collected in a pre-blink baseline window (−900 ms to −725 ms) to two components following the blink at intervals of 50–150 ms for the first component (C1) and 151–400 ms for C2 in temporal and parietal electrodes. To approximate previous studies, a subsample of younger participants (aged 17–50) in low workload conditions was used in the baseline analysis. The mean amplitude for the C1 component at the left and right parietal electrodes as well as in the right temporal electrode were significantly different from baseline. Moreover, at both the parietal and temporal regions, the mean amplitude for the C2 component was also significantly different from the mean baseline amplitude. These results regarding the deflection of the BRO response agree with previous studies examining BRO responses. For example, studies have found there to be a two-peak time-domain waveform present for the BRO phenomenon (Liu et al., 2017, 2020b). The two-peak phenomenon typically has one peak around the 100 ms latency and another around 300 ms (Liu et al., 2017, 2020b) similar to the latencies of the C1 and C2 components in the present work.

### 4.2 BRO responses, biology, and cognition

The results of this study showed modulations of the BRO response as a result of our workload conditions. No workload effects were found on the mean amplitude of the BRO C1 and C2 components at the left parietal and temporal electrodes. In contrast, workload did affect the BRO response in the right parietal region, such that in low workload conditions, the deflections for the C2 component were larger than in high workload conditions. Moreover, there was an effect of workload on both the C1 and C2 deflections in the right temporal regions such that in low workload conditions, there were larger C1 and C2 deflections as compared to the high workload conditions. These workload effects agree with laboratory-based studies examining the modulation of BRO responses based on mental load in relation to mental arithmetic tasks, which found reduced amplitudes of the second component (e.g., the positive going deflection at latencies between 80 and 170 ms for the C1 deflection and 280–420 ms for the C2 deflection) (Liu et al., 2020b). The workload modulation of the positive going deflection, without influencing the overall dynamics of the BRO waveform’s two peak component complex, is believed to be a result of competing visual task demands in differing workload conditions (Liu et al., 2020b). As a result of the processing of ongoing visual information during flight, there may be a reduced availability of neuronal resources for processing blink-related information as the brain is already engaged in ongoing visual tasks.

Previous work examining the BRO response has focused primarily on midline parietal electrodes due to their proximity to the precuneus region, which is a key cortical generator of the BRO response (Liu et al., 2020b). The present study used the lateral parietal and temporal regions to examine whether more accessible electrodes could capture workload effects on the BRO response. Although the precuneus is considered one of the major generators of the BRO response, BRO processing has also been associated with occipital, temporal, parietal and frontal regions, especially when workload effects were seen with tasks requiring higher order cognitive demands such as mental arithmetic (Liu et al., 2019). As such, it may be the case that the workload effects in this study, particularly in parietal and temporal regions, may be related to a competition of resources between blink-related processing and flight-related activity in these regions. Considering the navigation tasks integral to the present study, parietal regions may be particularly involved in spatial information processing and attention (Yantis, 2002; Shomstein and Yantis, 2006; Polich, 2007; Seydell-Greenwald et al., 2017; Liu N. et al., 2021). Pilots were also tasked with numerous communication tasks; thus temporal regions would also be highly involved in auditory information processing (Gandour, 2004; Meyer et al., 2005; Friederici, 2011).

The right parietal region of the brain is known to be involved in spatial attention processes (Yantis, 2002; Shomstein and Yantis, 2006; Seydell-Greenwald et al., 2017). These spatial cognitive abilities were assumed to be utilized by the study participants in both conditions, but more intensively in the high workload condition. Spatial processing is integral to take-offs, touch and gos, and planning circuit procedures (e.g., merging with traffic already in the circuit). Additionally, in our study the high workload conditions required substantial receptive and expressive radio communication. Pilots used the radio communication information to create and update mental models of the flight conditions. Substantial recruitment of parietal and temporal lobe regions associated with spatial and language functions would therefore necessarily occur during flight.

Seeing that the parietal and temporal lobe are implicated in aviator tasks (Polich, 2007; Käthner et al., 2014; Dehais et al., 2019), and that these brain regions were also where the BRO components were seen in the present work, the modulation of the BRO as a result of workload was expected. A neural compensation hypothesis may explain our results in the right parietal lobe. In particular the compensation-related utilization of neural circuits hypothesis (CRUNCH) theory (Reuter-Lorenz and Cappell, 2008) posits that as workload increases both younger and older adults recruit more neural circuitry, however, older adults will deplete their resources faster than younger adults, and performance on cognitive tasks will level off or drop as workload increases before the same reductions are seen with the younger adults. The CRUNCH impact on the BRO waveform can be seen in Figure 12, where at the C1 and C2 latencies, the difference between the peaks for low and high workloads was greater for the older as compared to the younger pilots. The younger participants’ C1 and C2 peaks were similar between workloads, but the older C1 and C2 peaks were greater in the high workload than in the low workload. Our results suggest that greater neural recruitment was necessary for older adults, even with lower task demands, and that a disproportionate change in the BRO peaks could be observed in high workload conditions in older adults.

In the right hemisphere, a different pattern of results was observed at the temporal as compared to the parietal electrode. This regional difference may be explained using a neural competition framework. As shown in Figure 15, at T8 both older and younger participants had greater peaks at C1 in the low, as compared to the high workload condition, although the younger participants show slightly more deflection in the high workload than is seen for the older participants. In the temporal region, where communication functions may be under particularly high demand in the high workload condition (numerous radio calls to attend to and to complete), both age groups exhibited less BRO at C1. This workload effect for both age groups indicates that ongoing recruitment for communication tasks may cause the circuitry normally acquired for the BRO to be otherwise engaged. Other work with pilots has shown that with high workload demands, the sensory-related stimulus response in ERPs (~100–200 ms) is attenuated in a central (albeit midline) brain region (Näätänen et al., 1988). Therefore, in high workload conditions, the C1 components might appear to be smaller as there is more neural competition in this region, and this might be affected at a sensory level.

The finding that the C1 deflection in the right temporal region was greater in low workload conditions as compared to high workload conditions has not been found in previous BRO studies. There is precedence in other research supporting our finding on attenuated sensory components. In auditory studies the N1 component, which occurs at similar latencies as the C1 component of the BRO waveform, is related to sound and feature detection in auditory cortex regions (Näätänen et al., 1988; Hyde, 1997; Trainor, 2007; Cox and Lakey, 2020). Thus, the first negative going peak in the BRO waveform might also be related to sensing and detecting new information (Liu et al., 2020b).

The results of this study also demonstrated that in the right parietal electrodes, age significantly affected the deflections of the components such that overall, older participants showed more positive-going deflections than younger participants. In the right temporal region, there was an interaction trend between age and workload such that there was a larger modulation of the BRO response in the older group as compared to the younger group. Prior work exploring BRO effects and aging demonstrates an inverted U-shaped relationship between age and BRO cortical activation across parietal and temporal regions, such that BRO effects increase with age until 71 years and then decrease beyond age 71 (Liu C. C. et al., 2021). This likely reflects greater cortical recruitment for BRO processing due to decreases in neural efficiency with aging and are consistent with the Scaffolding Theory of Aging and Cognition which states that the brain recruits additional resources as a compensatory mechanism for maintaining cognitive performance in the face of neurodegeneration (Oschwald et al., 2019). However, according to the theory, there is a limit to the amount of functional loss that can be compensated, and this is constrained by the availability of neuronal resources and the amount of neurodegeneration present in the older brain.

Our results also show a hemispheric asymmetry in that age and workload effects were observed in the right (P8 and T8) rather than the left hemisphere locations. This hemispheric asymmetry might be due to a technical reason, namely, the specific montage used in this study. Specifically, the reference electrode for the Emotiv headset used in this study is located at the P3 electrode location over the left hemisphere. As such, the lack of observed effects on the left hemisphere electrodes might be due to the removal of the signals and them being considered as common mode noise because of the proximity to the reference electrode.

Alternatively, the hemispheric differences observed in this study may reflect the underlying neuronal processing differences among hemispheres. Prior work has shown the functional specialization of the right parietal areas for processing the global spatial information necessary for accomplishing the flight tasks (Seydell-Greenwald et al., 2017) and the importance of the right temporal areas in spatial processing in aging (Ezzati, 2016). However, in both low and high workload flight conditions the left cortical structures would be heavily taxed with carrying out communication, visual, and motor tasks. Tasks frequently involved verbalizing flight information, and processing radio and visual information to maintain situation awareness. Regarding interference from motor activity, throughout the flight, all the participants operated the flight controls, including the throttle, flaps, and navigational aids with their right hand. As a result, the left motor and premotor cortex was highly involved in planning and carrying out the motor movements. With the left side of the brain playing such a significant role in flight-related tasks, it can be the case that the neural activity from the left side of the brain severely attenuated the BRO at the sensor level. This attenuation could ensue from a neural resource perspective, where fewer cortical resources were available to generate the BRO response during complex flight simulation. Or alternatively, the left side neural activity served as a “noisy’ background, through which the BRO was not easily detected.

Our findings are relevant to innovations in neurological testing where invisible-like EEG systems have the ability to capture neural signals during natural motion, therefore allowing for more real-world types of research (Bleichner and Debener, 2017). These highly portable systems use electrode locations around the ear, particularly on areas that are free of hair, including the mastoid bone (Bleichner and Debener, 2017). Additionally, researchers have also recently pioneered novel signal processing approaches that enable the use of such highly portable, low-density systems in realistic settings by enabling signal denoising (Hajra et al., 2020a) and improving signal capture (Hajra et al., 2021b) from EEG generally as well as specifically using BRO signals (Liu et al., 2019). Thus, our results from the temporal regions of the scalp are particularly encouraging and support the continued use of the BRO in measuring mental workload in useful and realistic settings.

### 4.3 Caveats and future directions

This work is the first study to investigate BROs using a low-density EEG sensor system in a simulated flight environment. Future work can involve greater dichotomization of workload, as in the present study the low workload condition still required significant cognitive demands. Limiting communication and motor activity could permit the detection of the BRO in the left hemisphere. To validate the findings in this study, further investigations can use additional electrodes, specifically at midline locations. Additional investigations into the effects of age on the BRO response should also be conducted with adults beyond the age of 70 and with a greater number of females to confirm and extend the present findings.

## 5 Conclusion

This study was the first to investigate blink-related oscillations under realistic settings using a portable, wireless, and easy-to-use EEG system. It was confirmed that a low-density EEG system can capture the BRO phenomenon in temporal and parietal regions, with significant increases in mean amplitudes in C1 and C2 deflections as compared to a baseline reference. Moreover, it also showed that workload and age differences in realistic settings modulated the BRO response. These findings represent a significant step forward in advancing the scientific understanding about the BRO phenomenon by elucidating its effects in aging, and also demonstrate its potential utility as a tool for evaluating cognitive performance in complex, realistic settings like the aircraft cockpit.

## Data availability statement

The data analyzed in this study is subject to the following licenses/restrictions: data are not publicly available due to ethical approval constrains. Requests to access these datasets should be directed to SGH, shajra@fit.edu.

## Ethics statement

The studies involving humans were approved by the Carleton University Research Ethics Board. The studies were conducted in accordance with the local legislation and institutional requirements. The participants provided their written informed consent to participate in this study.

## Author contributions

AZ: Formal analysis, Investigation, Writing – original draft, Writing – review & editing. KB: Conceptualization, Data curation, Formal analysis, Investigation, Methodology, Software, Supervision, Validation, Visualization, Writing – original draft, Writing – review & editing. CL: Conceptualization, Formal analysis, Software, Visualization, Writing – review & editing. CH: Writing – review & editing. SGH: Conceptualization, Formal analysis, Funding acquisition, Investigation, Methodology, Project administration, Resources, Software, Supervision, Validation, Visualization, Writing – review & editing.
